# Genomic regions on chromosome 5H containing a novel QTL conferring *barley yellow dwarf virus*-PAV (BYDV-PAV) tolerance in barley

**DOI:** 10.1038/s41598-019-47820-2

**Published:** 2019-08-05

**Authors:** Hongliang Hu, Shormin Choudhury, Sergey Shabala, Sanjiv Gupta, Meixue Zhou

**Affiliations:** 1 Tasmanian Institute of Agriculture, University of Tasmania, Private Bag 1375, Prospect, TAS 7250 Australia; 2 College of Science, Health, Engineering and Education, Murdoch University, Murdoch, 6150 Australia; 3 Plant Pathology, Department of Primary Industries & Regional Development WA, 3 Baron Hay Court, South Perth, 6151 Australia

**Keywords:** Biotic, Genetic markers

## Abstract

Barley yellow dwarf virus is a widespread disease affecting plant growth and yield in cereal crops including barley. Complete resistance to BYDV encoded by a single gene is lacking in barley. To identify novel resistance genes that can be further utilised in breeding for plant disease resistance, a doubled haploid population originated from a cultivated barley with a known resistance gene and a wild barley was constructed and assessed for barley yellow dwarf tolerance in three trials with two in Tasmania (TAS) and one in Western Australia (WA). We identified two Quantitative trait loci (QTL) in both Tasmanian trials, and four QTL in Western Australian trial. Two QTL from TAS trials were also detected from WA. The QTL on chromosome 3H corresponds to the known major resistance gene *Ryd2*. The other QTL, *Qbyd-5H*, represents a potential new resistance locus and contributed 7.0~10.4% of total phenotypic variation in the three trials. It was mapped within the interval of 125.76~139.24 cM of chromosome 5H. Two additional minor effect QTL were identified on chromosome 7H from WA trial, contributing slightly less effect on BYD tolerance. The consistently detected new gene on chromosome 5H will potentially serve as a novel source of tolerance to achieve more sustainable resistance to BYDV in barley.

## Introduction

*Barley yellow dwarf virus* (BYDV) is one of the most widespread and economically relevant plant viruses affecting a range of cereal crops, mainly barley, wheat, rye, oat and maize^1^. The phloem-limited virus is not mechanically or seed transmissible but is transmitted by one or more aphid species in a persistent, circulative manner^2^. Infection of BYDV causes many agronomical, biochemical, physiological and ultrastructural changes^3,4^, leading to major symptoms of leaf yellowing, stunted plant growth, which are known as barley yellow dwarf (BYD) disease^5,6^. Consequently, significant yield losses or reduction of yield components with oats showing the highest susceptibility of crop plants have been reported^7,8^. The disease is usually caused by one or a complex of closely related virus species. According to International Committee on Taxonomy of Viruses (ICTV), there are seven BYDV species belonging to different or unassigned genera in the family *Luteoviridae*. These include *barley yellow dwarf virus* (BYDV)-PAV, BYDV-MAV, BYDV-PAS, BYDV-Ker II, BYDV-Ker III, BYDV-SGV, BYDV-GAP. Among these, the most prevalent and economically important one is BYDV-PAV^9^. It is also one of the most commonly detected yellow dwarf viruses in Australia^10^. BYDV-PAV is transmitted most efficiently by both *Rhopalosiphum padi* and *Sitobion avenae*^11,12^.

Although field managements such as modifying sowing date to escape the disease and insecticide application to control aphid population are effective in limiting the spread of BYDV^13,14^, breeding and growing resistant varieties remains the most promising approach to address the challenge due to its inherent advantage of lower cost of management, durability of resistance and environmental friendliness^15^. Plant resistance to BYDV is usually divided into two types. The first is called virus resistance, which is characterised by reduced titre and restricted multiplication of virus in plant tissue as plant responds to virus infection. The second type is field resistance (tolerance), which represents mild or undiscernible symptoms of leaf discoloration and reduced loss of yield parameters, regardless of virus titre^16^.

Up to date, no complete resistance to BYDV encoded by a single gene is available in barley, though some partial resistance genes have been reported. The first reported partial resistance gene, *ryd1*, originated from the cultivar ‘*Rojo’*^17^. This gene provides very little resistance and has been rarely used in breeding programs. The second resistance gene, *Ryd2* was identified in Ethiopian landraces^18^. It has been introgressed into high-yielding barley cultivars, delivering much higher level of tolerance^19,20^. This gene is located on chromosome 3H, with possible existence of multiple alleles^21^. The mechanism of resistance of *Ryd2* may partly be attributed to its ability to reduce virus titre in BYDV-PAV or –MAV infected young plants^22^. However, this effect was not observed in old plants^23,24^. Many markers have been identified for the screening of *Ryd2*^5^. The third resistance gene, *Ryd3*, also originating from Ethiopian barley, was mapped on the centromeric region of chromosome 6H^25^. Ten tightly linked markers which co-segregate with *Ryd3*, were identified using high-resolution mapping^26^. Multiple studies also identified QTL associated with resistance/tolerance to BYDV-PAV^27–29^, located on chromosome 1H, 2H, 4H and 7H, respectively. Additionally, several QTL conferring tolerance to different yellow dwarf virus species have also been identified. del Blanco *et al*.^30^ identified two major QTL for CYDV-RPV tolerance on chromosomes 2H (*Qcyd*.*MaBu-1*) and 7H (*Qcyd*.*MaBu-2*), and 4 minor QTL on chromosomes 3H, 4H, and 2H were identified from a population of recombinant inbred lines constructed with Madre Selva and Butta 12. QTL for tolerance against Illinois isolate BYDV-PAV was also identified using 428 spring oat lines. Six significant single nucleotide polymorphism (SNP) marker-trait associations representing two QTL were found on chromosomes 3C (Mrg17) and 18D (Mrg04)^31^.

Despite this, the current constraint of available resistance/tolerance genes, especially of highly effective ones, still constitutes a major impedance to the breeding effort focusing on the improvement of BYD resistance. Exploration of novel genes conferring robust BYD resistance is necessary and a priority in breeding programs. With the aim of identifying novel genes responsible for BYDV-PAV tolerance/resistance, we, herewith, report quantitative trait loci (QTL) with tolerance trait of leaf yellowing using a doubled haploid (DH) population of barley.

## Results

### Frequency distribution of BYD symptoms

**Figure 1:**
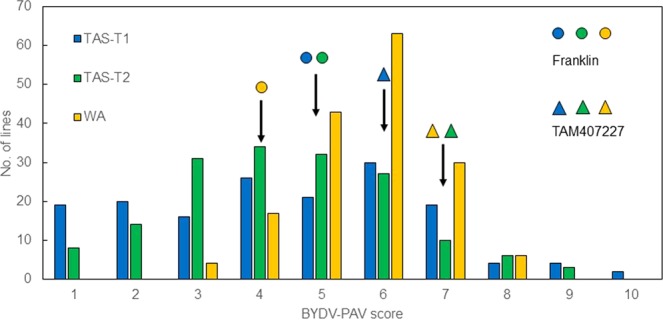
Frequency distribution of symptom scores for BYDV-PAV infected doubled haploid barley lines derived from TAM407227 and Franklin. Number of lines corresponding to score value ‘1’ means the number of lines with averages in the interval from 0–1, and so forth. Circles with different fill types indicate scores of Franklin in different trials; Triangles of different fill types indicate scores of TAM407227 in different trials.

In TAS-T1& T2 trials, symptoms of virus infection started to appear two weeks after inoculation. The frequency distribution of BYD symptoms scored based on leaf yellowing showed that the phenotype in both TAS and WA trials basically followed nearly normal distribution (Fig. 1). Both parent varieties showed moderate tolerance to BYDV-PAV, with scores of 5 and 6 for Franklin and TAM407227, respectively. The population sown in WA developed severer symptoms overall than in both TAS trials, with the scores of most lines clustering between 5 and 7. Both genotype and environment (sowing time and location) showed significant effects on symptom scores for the DH lines (Table 1), with the effect of genotype being more significant than environment.

**Table 1:** ANOVA test of BYDV symptom score across three trials/environments in 2017.

Source of variation	df^a^	Sum of squares	Mean square	F value	Pr > F
Genotype	98	1858.3	19.0	11.4	<0.0001
Environment	2	846.1	423.1	254.44	<0.0001
Genotype × Environment	196	1710.3	8.7	5.25	<0.0001
Error	1078	1792.4	1.7		

^a^Degree of freedom.

### Correlation analysis between different years

**Figure 2:**
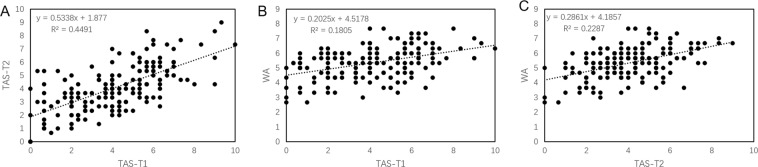
Correlation analysis of BYD symptom score in three trials conducted under two environments (**A)**. TAS-T1 and TAS-T2 (**B)**. TAS-T1 and WA (**C)**. TAS-T2 and WA).

The scoring data of each year was used for correlation analysis. Significant correlations values were found between TAS trials, with TAS-T1 and TAS-T2 showing the highest R^2^ (0.4491) (Fig. 2A). TAS and WA trials showed weaker correlations (R^2^ = 0.18 (TAS-T1 vs WA) and 0.23 (TAS-T2 vs WA) Fig. 2B,C) indicating additional/different genes operative in WA environment.

### QTL for BYD resistance

**Figure 3:**
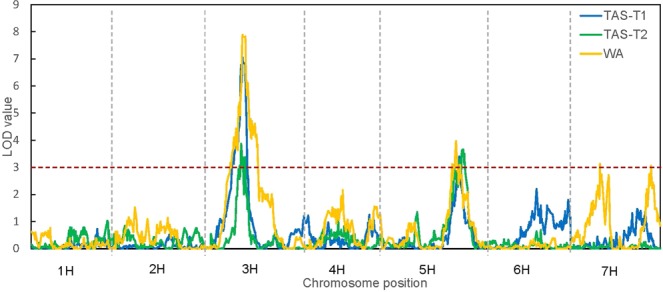
LOD scan of restricted MQM (rMQM) mapping across seven chromosomes for QTL conferring BYDV-PAV tolerance in TAS and WA. TAS-2: 17 dpi in Tasmania Trial; TAS-3: 19 dpi in Tasmania Trial; WA: Western Australia Trial.

**Figure 4:**
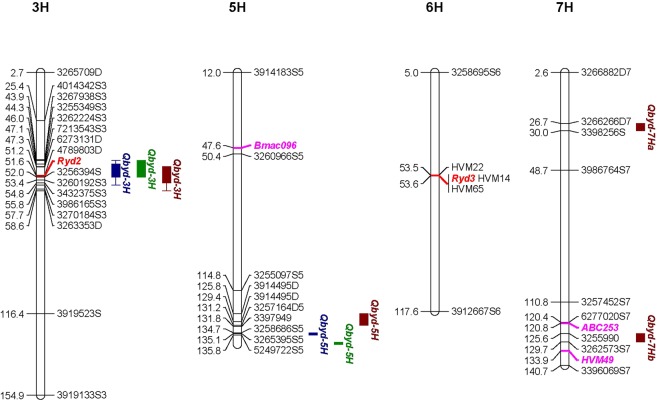
Genetic positions of identified QTL and genes responsible for BYDV-PAV tolerance in Morex consensus map. QTL are represented by confidence intervals with LOD attenuation of 1 (closed box) and 2 (closed), respectively. Colours of QTL indicate different observations (Blue: TAS-T1; Green: TAS-T2 Brown: WA;). Markers in pink indicates associated QTL for BYDV resistance in other studies.

QTL analysis was performed using symptom scores at the date of symptom manifestation for each trial. Results of QTL analysis using a multiple QTL mapping (MQM) approach showed that tolerance to BYDV-PAV was attributed to multiple loci. The confidence interval of detected QTL was determined by locating the nearest flanking marker on each side of the peak marker with LOD attenuation of 2 (2-LOD interval to obtain a 95% confidence interval). Figures 3 and 4 and Table 2 show the QTL identified from different data sets. Two chromosomal regions were consistently detected for BYDV-PAV tolerance in both TAS trials whereas in WA trial, four QTL were detected.

**Table 2:** List of SNP and DArT markers significantly associated with trait leaf yellowing.

Trial^a^	Marker^b^	Chr^c^	Pos^d^ (cM)	LOD^e^	R^2^(%)^f^	Add^g^	Interval^h^ (cM)	Gene^i^	Parent^j^
TAS-T1	3262224S3	3H	46.03	6.6	14.6	1.27	44.3–55.8	*Ryd2*	Franklin
	3258686S5	5H	134.72	4.9	8.4	−0.82	131.2–135.8		TAM407227
TAS-T2	3262224S3	3H	46.03	3.9	11.0	0.80	44.3–52.0	*Ryd2*	Franklin
	4789985D	5H	139.24	3.7	10.4	−0.70	139.1–139.2		TAM407227
WA	4789803D	3H	51.20	7.9	14.6	0.43	47.1–58.6	*Ryd2*	Franklin
	3263957S5	5H	129.44	4.0	7.0	−0.29	125.8–131.2		TAM407227
	5249736S7	7H	29.82	3.1	6.5	0.24	26.7–30.0		Franklin
	3257158S7	7H	126.56	3.1	6.3	0.24	125.6–129.7		Franklin

^a^Location of trial. TAS-T1, TAS-T2: Launceston, Tasmania; WA: Manjimup, Western Australia.

^b^Peak marker most significantly associated with trait.

^c^Chromosome of detected peak marker.

^d^Genetic position of peak marker in barley Morex consensus map.

^e^LOD value for peak marker.

^f^Percentage of explanation for total phenotypic variance.

^g^Additive effect, indicating the effect by parent TAM407227.

^h^Interval position of the putative QTL, determined by rMQM, with LOD > 3, in cM on the genetic map^i^ Putative gene corresponding to QTL.

^i^Correspond^i^ng genes for identified QTL based on comparison of genetic positions.

^j^Parent contributing favourable allele at the locus.

In TAS-T1, the major QTL (*Qbyd-3H*) on chromosome 3H was mapped to 46.0 cM with the identified marker 3262224S3, corresponding to Morex contig 44719. The 2-LOD flanking markers were 3255349S3 and 3986165S, determining a confidence interval from 44.3 to 55.8 cM. This locus explained 14.6% of total phenotypic variation. The second QTL (*Qbyd-5H*) was located within the interval from 131.2–135.8 cM on chromosome 5H, explaining 8.4% of the phenotypic variation.

In TAS-T2, the 3H QTL, *Qbyd-3H*, was also associated with the peak marker 3262224S3 (46.0 cM). The flanking markers for 2-LOD interval were 3255349S3 and 3914642D, corresponding to 44.3 and 52.0 cM. This QTL explained 11% of the phenotypic variation. The other marker, 4789985D, was detected on chromosome 5H with a similar position to that identified from TAS-T1. It was mapped to 5HL within a confidence interval flanked by two markers 4016391S5 (139.1 cM) and 3255873S5 (139.2 cM) and explained 10.4% of the phenotypic variation. The peak marker 4789985D is located at 139.2 cM and corresponds to Morex genome assembly contig 244441.

Four QTL were detected in WA trial. The major effect QTL (*Qbyd-3H*), which explained 14.6% of the phenotypic variation, was located on chromosome 3H at a same position as that identified from TAS trials. The QTL on 5H (*Qbyd-5H*), accounting for 7.0% of the phenotypic variation, was also located at a similar position to that identified from TAS trials with an interval of 125.7~131.1 cM. Two additional QTL were identified from WA trial, both located on chromosome 7H. *Qbyd-7Ha* had an interval of 26.7~30.0 cM and explained 6.5% of the phenotypic variation and *Qbyd-7Hb* was located at 125.6~129.7 cM and explained 6.3% of the phenotypic variation. Franklin contributed tolerance alleles for both loci.

### Additive effects of QTL

**Figure 5:**
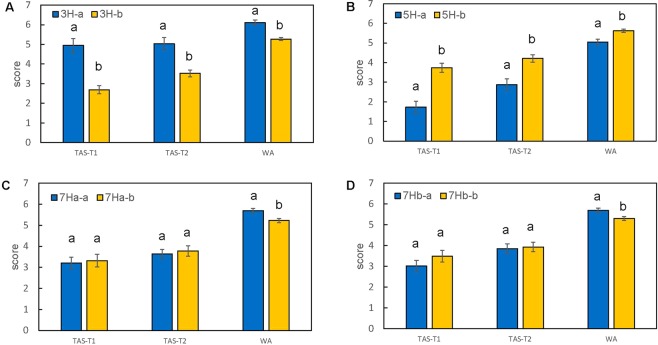
Mean values of DH lines groups based on peak marker calls (a/b) of identified QTL for the three trials. (**A**) *Qbyd-3H* (**B**) *Qbyd-5H* (**C**) *Qbyd-7Ha* (**D**) *Qbyd-7Hb*. Bar represents the mean ± standard error of the mean. Different letters within each trial indicates significant difference between different marker calls at P < 0.05.

Although slightly different markers were identified in different trials, especially for QTL on chromosome 3H and 5H, we found the similarly positioned markers had basically identical marker calls across the 163 DH lines (Supplementary Table 1). Based on the marker types (*A/B*) of *Qbyd-3H* for the 163 DH lines, we assigned the DH lines to two groups, and found the group of marker type *B*, which is the marker type for Franklin, has a significantly lower mean value than the group of type *A* (Fig. 5), suggesting that the tolerance allele is derived from Franklin. Similarly, for *Qbyd-5H*, the mean value of type *A*, which is the marker type for TAM407227, was significantly lower than type *B* (Fig. 5), suggesting the tolerance allele for *Qbyd-5H* is derived from TAM407227.

**Figure 6:**
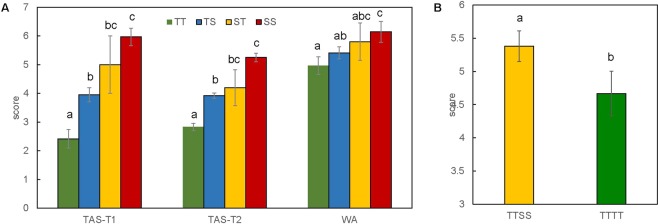
Average score for BYDV-PAV tolerance of different combinations of barley lines. (**A**) Barley lines was divided into four genotypes based on the polymorphism of the markers: 3262224S3 (*Qbyd-3H*), 3258686S5 (*Qbyd-5H*). The first letter of genotype represents the tolerance (T)/susceptibility (S) allele of *Qbyd-3H* and the second represents the T/S allele of *Qbyd-5H*. (**B**) Barley lines in WA trial was divided into two types differering in marker calls on *Qbyd-7Ha* and *Qbyd-7Hb* but was identical at *Qbyd-3H* and *Qbyd-5H*. The first letter represents the T allele of *Qbyd-3H* and the second represents the T allele of *Qbyd-5H*. The third letter represents the T/S allele of *Qbyd-7Ha* and the fourth represents the T/S allele of *Qbyd-7Hb*. Numbers on top of columns indicate the number of lines for each type of combination. Bar represents the mean ± standard error of the mean. Different letters within each trial indicates significant difference between genotypes at P < 0.05.

The two major QTL identified on 3H and 5H showed additive effects. The 163 DH lines were grouped into four groups according to their base calls of the marker 3262224S3 (*Qbyd-3H*) and 3258686S5 (*Qbyd-5H*) and the average score of each group was calculated (Fig. 6A). Among the four types of combination, the combination of tolerance allele for *Qbyd-3H* and *Qbyd-5H* (TT) showed the highest level of tolerance. The mean score for TAS-T1, TAS-T2 and WA trial was only 2.4, 4.9 and 2.8, respectively. In contrast, the combination of susceptible allele for *Qbyd-3H* and *Qbyd-5H* (SS) showed the lowest level of tolerance, with the mean value from different trials ranging from 5.2 to 6.2. The additive effect of the two 7H QTL in WA trial is shown in Fig. 6B. The addition of two 7H tolerance alleles, combined with tolerance alleles from *Qbyd-3H* and *Qbyd-5H* (TTTT) showed slightly better tolerance (average score of 4.6) than those with only tolerance alleles from 3H and 5H (TTSS, average score of 5.3).

## Discussion

The traditional approach of identifying virus resistant genotypes usually involves aphid inoculation, disease scoring and ELISA test for virus titre, which can be both time consuming and labour intensive. Development of molecular markers associated with disease resistance is a more powerful and effective tool as it enables rapid screening of elite genotypes and thus accelerates the breeding program.

Considering barley yellow dwarf as a severe and proliferated plant disease in many regions of the world, it will be of high significance to identify high quality markers associated with novel genes conferring BYD resistance/ tolerance. However, evaluation of plant resistance to BYDV is complicated by numerous factors such as aphid specificity and availability, field climate which affects aphid transmission and propagation, and plant growth stage when aphid feeding occurs^3,32–35^. It is therefore necessary to screen for BYD resistance under multiple environments. In this study, we used two locations differing in field climate and aphid prevalence to achieve a more reliable assessment of BYDV-PAV tolerance. Although the plants in Tasmanian trials were artificially inoculated, higher degree of disease severity and disease incidence was observed in Western Australia, where two additional QTL were detected. As increased number of aphids was associated with higher rate of infection^3^, more aphids were used in TAS trials to ensure the infection of all the lines. Furthermore, the WA trial scored severer overall symptom and the correlation values of TAS and WA trials being not very high, which indicates different genes operative under different environments. Given that we have repeated phenotyping for BYD resistance in TAS, it is more likely there exist more potential genes such as *Qbyd-7Ha* and *Qbyd-7Hb* being operative in WA.

The identification of multiple QTL conferring BYDV-PAV tolerance reflects large genetic differences between the parents. Two QTL were detected in both locations. The interval for *Qbyd-3H* corresponds to the known resistance gene *Ryd2*^36,37^, according to the position of markers linked to *Ryd2*^25,38,39^. By locating the flanking markers for *Qbyd-3H*, the candidate genes for this locus is limited to 113 (Supplementary Table 2), with a corresponding physical position of 499457724 bp -514835085 bp. A possible candidate gene is HORVU3Hr1G067050 which expresses leucine-rich repeat (LRR) receptor-like protein kinase family protein. LRR receptor-like protein kinase is a type of cell surface receptors represented by receptor(-like) kinases (RKs, RLKs), which consists of extracellular receptor, transmembrane and intracellular protein kinase domains^40^ and is involved in plant pathogen defence^41^. *Ryd2* not only confers field tolerance as observed in this study, it has also been reported to be capable of reducing virus titre in young plants infected with BYDV-PAV or BYDV-MAV^42,43^. *Ryd2* has been reported to possess dominant or minor effects, possibly for genetic reasons^28^. The specific gene function and the mechanistic role of *Ryd2* in expressing plant resistance/ tolerance to BYDV are still unknown and warrants further characterisation.

**Figure 7:**
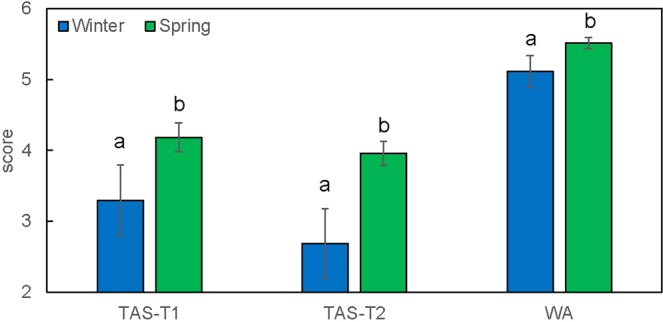
Average scores for BYDV-PAV inoculated spring- and winter-type lines grown in TAS and WA trial. Bars indicate standard errors. Different letters within each trial indicates significant statistical difference at P < 0.5. Bar represents the mean ± standard error of the mean.

**Figure 8:**
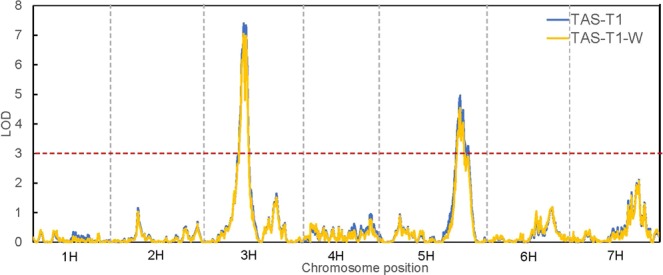
Comparison of LOD scan curves of interval mapping results. TAS: results generated by using TAS-2 scores; TAS-2-W: results generated by using winter-type habit as covariate.

Another QTL, named *Qbyd-5H*, was consistently detected on chromosome 5H in all three trials. *Qbyd-5H* is the second major QTL given its percentage of explanation for phenotypic variance and additive effect. Combining the intervals of QTL identified from different trials, this QTL fell in the interval of 125.6 cM to 135.8 cM, covering a total of 476 high confidence genes (Supplementary Table 3) located at 600469098 bp - 623964472 bp on the corresponding physical map. Three genes are functionally annotated as disease resistance genes (HORVU5Hr1G096560, HORVU5Hr1G096620, HORVU5Hr1G096630) and thus can be the candidate genes for *Qbyd-5H*. Other candidate genes within the region include: HORVU5Hr1G099940 (disease resistance protein), HORVU5Hr1G102250 (disease resistance protein). A putative QTL on 5H for relative number of ears per plant after BYDV-PAV infection was identified in two different populations^28^ but the position of associated marker (Bmac096, 47.64 cM) is different from the QTL identified in this study. Interestingly, the vernalisation gene, *Vrn-H1* which encodes a MADS-box transcription factor (HORVU5Hr1G095630), is localised within the interval of *Qbyd-5H*. The spring barley lines scored significantly (P < 0.05) severer symptom than winter barley lines in all the three trials (Fig. 7). However, when using the winter growth habit as a covariate in QTL analysis, the QTL for BYD resistance was unchanged (Fig. 8), indicating that *Qbyd-5H* represented a disease resistance gene positioned closely to *Vrn-H1* but not pleiotropic.

The two putative QTL on chromosome 7H were only detected in WA trial which showed severer symptom but contributed relatively less to total phenotypic variation than *Qbyd-3H* and *Qbyd-5H*. *Qbyd-7Hb* (125.6–129.7 cM) is co-located with the previously reported QTL conferring resistance against BYDV-MAV and BYDV-PAV^29^. However, the reported one explained a greater percentage of the phenotypic variation (18~20%) than the current QTL (6.3%), depending on the virus serotypes. There are 28 and 41 genes within the interval of *Qbyd-7Ha* and *Qbyd-7Hb*, respectively (Supplementary Tables 4 and 5). *Qbyd*-7Ha covers a region from 38096969 bp to 40087264 bp whereas *Qbyd-7Hb* ranges from 640327581 bp to 641757816 bp. Two disease resistance genes (HORVU7Hr1G114850, HORVU7Hr1G114880) are located within the interval of *Qbyd-7Hb*.

Although resistance genes are very abundant in plant genome, historically the lack of naturally occurring genes conferring strong BYD resistance has been the major obstacle to development of varieties with durable resistance to the widespread disease. Both of major effect BYD resistance genes, *Ryd2* and *Ryd3*, were identified from Ethiopian barley^25^, a proved valuable gene pool for BYD resistance or tolerance^18^. A qualitative gene, *Ryd4*^*Hb*^, was introgressed from *Hordeum bulbosum* into barley to confer dominant resistance to BYDV^44^. Wild barley is a highly useful germplasm with a wide genetic diversity as it contains rich source of elite alleles^45^, compared with cultivated barley^46^. In this study, the tolerance allele for *Qbyd-5H* is originated from the wild barley parent, TAM407227. This allele is likely to provide barley breeders with a new alternative to achieve more complete and robust resistance to combat the widespread and devastating disease.

## Conclusions

In conclusion, we identified a total of four QTL for BYDV-PAV tolerance. The large effect QTL, *Qbyd-3H* coincides the known major effect gene *Ryd2*. Another major QTL, *Qbyd-5H*, represents a novel locus which should be useful in future breeding due to its significant effect. The novel QTL offers more robust and sustainable tolerance sources to breeders. No QTL was detected approximate to the position of *Ryd3*, which was located at 54.8 cM on chromosome 6H based on the marker Bmac0018. The combination of this QTL with other genes, such as *Ryd2* and *Ryd3*, can greatly improve the tolerance to BYDV of barley cultivars. Further work such as fine mapping is required to understand from the genetic, molecular and physiological mechanisms of *Qbyd-5H*.

## Methods

### Mapping population

A total of 163 lines from a DH population of barley derived from the cross of Tam407227 (*Hordeum spontaneum*) and Franklin (*Hordeum vulgare* L.) with a pedigree of Shannon/Triumph was used in this study. Wild barley Tam407227 was introduced from Australian Grains Genebank and Franklin is an Australian malting barley variety, containing resistance gene *Ryd2*^47^. This population was originally used for identifying QTL for waterlogging tolerance as that two parents showed significant difference in the tolerances^48–50^ but later we observed segregation in BYDV resistance in our preliminary trial.

### Viruliferous aphid propagation

*Rhopalosiphum padi* aphids were collected from the field at Mt Pleasant Laboratories, Prospect, Tasmania, Australia (147°08′E, 41°28′S). The aphids were later transferred to inoculate young plants using susceptible wheat *var*. Mace in a glasshouse with temperature controlled favourable for aphid. To validate the existence of BYDV-PAV, plant leaves of symptomatic plants were sampled and tested by enzyme-linked immunosorbent assay (ELISA). Plants were regularly renewed to ensure enough aphids to be used to inoculate the population. qPCR and semi-quantitative PCR showed that only a high concentration of BYDV-PAV was detected in susceptible host. The nucleotide sequence (GenBank: MK962883)^51^ showed 96.64% similarity with the Australia isolate (GenBank: X07653.1)^52^.

### Glasshouse and Field trial in Tasmania

Barley seeds of 163 lines were sown in small pots using potting mix medium incorporated with fertiliser in September and November 2017 for the 1st trial (TAS-T1) and the 2nd trial (TAS-T2), respectively. Three seeds per line were sown in each pot. Three replicates were applied. For BYDV-PAV treatment, each line was inoculated with viruliferous aphids restricted in a breathable mesh container clipped on leaves to avoid aphids escape^3^. One week after inoculation, the clips were removed, and insecticides were sprayed to kill aphids. Barley seedlings were transplanted to cultivated plots in the field at Mt Pleasant laboratories. Slow release fertiliser was applied after plants were transferred to the field. Supplementary irrigation using sprinkler was used, lasting one hour at middle of the day on a daily basis. Fungicide was sprayed every two weeks after seed germination.

### Field Trial in Western Australia

The screening trial was also conducted in 2017 at Regional DPIRD Field Station, Manjimup, Western Australia (116°08′E, 34°12′S) where the occurrence of BYDV and CYDV, along with respective vector aphids, have been reported to prevail regularly^10,53^. Each line was sown in duplicate, where twenty seeds were directly sown in a row length of 1.2 m. The trial was exposed to aphids (after its multiplication on oat plants) under natural infection at the site.

### Scoring of disease severity

For each trial, plant response to BYDV-PAV was recorded at anthesis stage (GS 60–70) when symptoms of infection are fully displayed. A standardized disease scoring system was used to assess the level of virus resistance based on foliar discoloration as reported by Saari and Prescott^54^, with minor adaptions for barley.

### Map construction

Diversity Arrays Technology (DArT) and SNP markers were developed and conducted by Triticarte Pty. Ltd. A rich abundance of polymorphism was identified, generating around 15,000 DArT markers and 14,500 SNP markers polymorphic between Franklin and TAM407227. Markers with more than 10% missing data and duplicate markers (markers located at same/similar positions) were deleted, leading to a final number of 5626 high-quality markers used for QTL analysis.

### QTL analysis

The symptom scores were used for QTL mapping. The data was first analysed by interval mapping (IM). A screening threshold of logarithm of the odds (LOD) value of 3.0 was used to determine the presence of a QTL. The marker with the highest LOD value at each putative QTL identified using interval mapping (IM) was selected as a cofactor and were used as genetic background control in the approximate multiple QTL model (MQM) analysis of MapQTL6.0 (Kyazma, Japan). The results were later subjected to restricted MQM (rMQM) mapping which does not use markers close to the QTL as cofactors. Two markers which had LOD attenuation of 2 were selected as flanking markers to define the confidence interval of each QTL^55^. The percentage of variance explained by each QTL (R^2^) was obtained from rMQM mapping. Graphic illustrations of the linkage groups and detected QTL with associated markers were generated using the program MapChart 2.2^56^.

### Functional annotations of genes

To compare with known BYDV resistant genes, the genetic position of QTL in this study were all converted to genetic positions in Morex consensus map location using the peak marker sequence to blast at IPK barley blast server (http://webblast.ipk-gatersleben.de/barley_ibsc/). The blast hit with the highest match was selected as the most likely contig corresponding to the peak marker. The physical position and functional description of high confidence genes within the confidence interval of identified QTL were also generated. Annotated functions in barley were downloaded from http://webblast.ipk-gatersleben.de/barley_ibsc/downloads/.

### Statistical analysis

Data analysis for the distribution frequency of BYDV score was performed with the SPSS 19.0.0 (IBM Corporation, Armonk, NY). Analysis of variance (ANOVA) was applied using the symptom scores of DH lines following BYDV-PAV infection across three trials to assess the effect of different sources of variation, including environment, genotype and genotype × environment. All data is expressed as mean ± standard error. Student’s t-test was used for statistical evaluations using SPSS 19.0.0.

## Supplementary information

supplementary legends

Table S1

Table S2

Table S3

Table S4

Table S5

## Data Availability

The datasets generated during and/or analysed during the current study are available from the corresponding author on reasonable request.
