# Supplementary material for: Genomic regions on chromosome 5H containing a novel QTL conferring barley yellow dwarf virus-PAV (BYDV-PAV) tolerance in barley
Source: Sci Rep. 2019 Aug 5;9:11298. doi: 10.1038/s41598-019-47820-2 (PMC6683297; doi:10.1038/s41598-019-47820-2)
Supplement: Supplementary file 1 — supplementary legends [file 41598_2019_47820_MOESM1_ESM.docx]

**Genomic regions on chromosome 5H containing a novel QTL conferring barley yellow dwarf virus-PAV (BYDV-PAV) tolerance in barley**

Hongliang Hu^1^, Shormin Choudhury^1^, Sergey Shabala^1^, Sanjiv Gupta^2,3^, Meixue Zhou^1^

**Supplementary information**

Table 1. Comparison of Marker calls of identified QTL for BYDV-PAV tolerance. Different markers were identified from different trials representing the same QTL. XLSX 14 KB

Table 2. Candidate high confidence genes for *Qbyd-3H*. XLSX 44 KB

Table 3. Candidate high confidence genes for *Qbyd-5H*. XLSX 37 KB

Table 4. Candidate high confidence genes for *Qbyd-7Ha*. XLSX 11 KB

Table 5. Candidate high confidence genes for *Qbyd-7Hb*. XLSX 12 KB
